# Virtual Reality Use Among Persons With Dementia: An Integrative Review

**DOI:** 10.1093/geroni/igaa057.3000

**Published:** 2020-12-16

**Authors:** Justine Sefcik, Darina Petrovsky, Pamela Cacchione, Sungho Oh, George Demiris

**Affiliations:** 1 Drexel University, Philadelphia, Pennsylvania, United States; 2 University of Pennsylvania, Philadelphia, Pennsylvania, United States

## Abstract

It is not well understood how virtual reality (VR) is currently used by older adults who have cognitive deficits due to dementia. The aim of this integrative review was to examine and report on published research exploring VR use among older adults with dementia. We searched 3 data bases for publications and used Whittemore and Knafl’s methodology for data extraction. Out of 122 articles we identified 24 that met our inclusion criteria, 15 published in 2012 and later. Most articles (12) used VR for assessment, and the others used VR for cognitive training (5) and as an intervention (3) (i.e., for exercise). Sample sizes were 30 or fewer persons with dementia. There is heterogeneity in the types of VR equipment, experiences, and foci of assessment through VR use. We identify opportunities to further explore VR as an intervention for persons with dementia to improve quality of life.

